# Prevalence of metabolic disease and correlation to body composition and cardiovascular fitness in adults undergoing fitness assessments

**DOI:** 10.1371/journal.pone.0209514

**Published:** 2018-12-21

**Authors:** LesLee Funderburk, Matthew Peterson, Kaitlan Beretich, Nish Shah, Peter W. Grandjean

**Affiliations:** 1 Robbins College of Health and Human Sciences, Baylor University, Waco, Texas, United States of America; 2 Orthopedics and Sports Medicine, Houston Methodist, Sugarland, Texas, United States of America; Temple University, Lewis Katz School of Medicine, UNITED STATES

## Abstract

The purpose of this descriptive study was to assess the prevalence of metabolic syndrome (MetS), prediabetes and type 2 diabetes (T2DM) in participants who voluntarily participated in a fitness assessment, and to examine associations with routine nutrition intake and overall body composition. One hundred and six participants were recruited. Anthropometric measurements were taken with blood analyses completed for fasting glucose, glycosylated hemoglobin (HbA1c) and lipid panel. A 24-hour diet recall and a dietary screening survey was used to assess nutrient intake, in a sub-set of 36 participants. Statistical analyses utilized partial Spearmans’ rank correlations, risk ratios, and Kendall’s Tau correlations, with significance level at p < 0.05. Twenty five percent of this sample had ≥ three risk factors for MetS, with elevated fasting glucose and blood pressure being the most prevalent. Twenty percent of the participants had HbA1c levels elevated at the prediabetes range, with no previous diagnosis. Four percent of participants had HbA1c levels elevated at the T2DM range. Two nutrients of interest were correlated to BMI status. Percent kcal from carbohydrate (τ -0.207, *p*<0.05) had a negative correlation with BMI status and percent kcal from fat intake had a positive correlation (τ 0.217, *p*<0.05). Findings from this small sample of adults indicate the need for routine assessment of: clustering of MetS risk factors, risk of prediabetes and T2DM and treatment of same. Many participants would benefit from increasing their participation in physical activity, weight loss in regard to overall health improvement, and education to improve diet quality.

## Introduction

Nearly 35% of U.S adults have metabolic syndrome (MetS) [1]. Metabolic syndrome is defined by the National Heart, Lung and Blood Institute (NHLBI) as a clustering of three or more of the following risk factors: central adiposity, elevated triglycerides, reduced high density lipoprotein (HDL) cholesterol, hypertension, and elevated fasting plasma glucose [2]. Some postulate that the more risk factors one has, the greater the chance of developing cardiovascular disease (CVD) and/or type 2 diabetes (T2DM) [3]. Of note, research has shown that as the number of risk factors increases, so does the risk of CVD-related mortality [4]. Sherling et al [5] suggests MetS may be the new silent killer due to the number of people either under-treated or undiagnosed.

One of the modifiable risk factors for CVD is physical activity (PA) [6]. Nationally, only one in four adults meet federal guidelines for both aerobic and muscle-strengthening activity, with approximately 32% meeting one guideline [7]. A key component of physical fitness is cardiorespiratory endurance, defined as “the ability to perform dynamic exercise involving large muscle groups at moderate-to-high intensity for prolonged periods” [8]. In adults who report engaging in routine PA, there is an inverse association in dose-response and mortality, in those with and without cardiovascular disease [9].

Glycosylated hemoglobin (HbA1c) is a long-term marker of blood glucose control and is independently associated with negative micro- and macro-vascular outcomes [10,11]. According to the American Diabetes Association (ADA) Standards of Medical Care in Diabetes (2016), prediabetes is diagnosed with a HbA1c of 5.7 to 6.4% and T2DM at ≥ 6.5% [12]. The prevalence of diabetes among U.S. adults was described in a review; the researchers found 9.4% of the population had diagnosed T2DM with 7.2% undiagnosed, and 33.9% with prediabetes [13].

Consuming a healthy, nutritionally balanced diet can help prevent or treat many chronic health conditions, including MetS [14]. According to the 2015–2020 Dietary Guidelines for Americans (DGA), many adults need to make shifts in their current intake to achieve a healthy eating pattern. Recommended shifts include increasing intake of whole grains, fruits, vegetables, and dairy and decreasing fat intake, in particular saturated fat [15].

Adults located in the central Texas region have the opportunity to participate in a curriculum-based community service program offered in collaboration with a local family medicine residency program. These assessments include a blood biochemical analysis, body composition assessments, and a general evaluation of the individual’s strength and cardiovascular fitness. The information is then provided to participants in the form of an individualized exercise prescription in hopes of helping them improve their physical fitness. Nutrition counseling, education, or screening is not currently offered to these clients.

Serum vitamin D insufficiency and deficiency were previously reported in a sub-sample of this study’s participants [16]. The purpose of this descriptive study was to further explore and assess the prevalence of metabolic disease among participants who voluntarily completed the various health assessments, and examine the associations among routine nutrition intake, PA, and overall body composition. This study can provide useful information for the design of future fitness evaluations and subsequent provision of nutrition education to promote improvements in lifestyle.

## Materials and methods

This was a cross-sectional, descriptive study approved by the Baylor University Institutional Review Board. One hundred and six participants were recruited between January 2017 and April 2018, which accounted for 75% of adults taking part in the fitness evaluations, exceeding the initial goal of 50% recruitment. Male and female volunteers arrived at the exercise laboratories after an 8- to 10-hour fast—limited to water ingestion only—with shorts, t-shirt, and a pair of comfortable jogging/running shoes. After providing written informed consent to participate, volunteers were asked to complete a Health History Questionnaire which was subsequently reviewed by a physician. Blood pressure measures and heart rate after 5 minutes of supine rest were obtained. Demographic variables include age, sex, and race/ethnicity. All data collection was completed at time of assessment appointment.

### Anthropometrics and body composition

All data collection occurred in person by trained individuals and included the following: age, gender, height, weight, waist circumference and visceral adipose tissue (VAT). Basic anthropometrics of height, weight, and waist circumference were measured with participants in exercise clothing, without shoes, but in stocking feet. Height was measured to the nearest 0.10 centimeter utilizing a stadiometer. Weight was measured to the nearest 0.10 kilogram on a calibrated, digital scale (Tanita, SC 331S). Body mass index (BMI) was calculated as weight in kilograms divided by height in meters squared. Standardized waist circumference was obtained using the NHLBI guidelines [17]. Body fat and VAT measures were obtained using the dual energy x-ray absorptiometry (DXA) bone densitometer (Hologic Discovery QDR, Bedford, MA).

### Exercise assessment

Routine exercise participation was assessed from self-reported number of exercise sessions, of any type, participants engaged in per week. This was then multiplied by the number of minutes per session and used as an indicator of routine exercise quantity. Once cleared by the attending physician, apparently healthy volunteers completed a standardized maximal graded exercise test on a treadmill to determine his or her cardiovascular fitness [8]. The treadmill test was continuous and progressed by increasing treadmill speed and grade every three minutes until the volunteer achieved volitional fatigue [18,19]. Trained laboratory technicians monitored heart rate, electrocardiographic tracings, blood pressure, and rating of perceived exertion (RPE) throughout the treadmill test. Hemodynamic responses to exercise of increasing intensity were characterized and cardiovascular fitness (VO_2max_) was estimated from treadmill test time [20,21].

### Blood biochemistry

The biochemical markers evaluated included a comprehensive metabolic panel, complete blood count with differential, HbA1c, and a lipid panel. All blood draws were completed by trained lab staff. The blood analyses were completed and reported by Clinical Pathology Laboratories (Waco, TX) utilizing Roche Cobas enzymatic colorimetric HK generation 3 for fasting glucose; Roche Cobas Integra immunoassay (TINA-QUANT HbA1c DX Generation 2) for HbA1c; and Roche Cobas enzymatic colorimetric generation 2 for the lipid panel.

### Dietary intake

To assess nutrition intake, a sub-sample of participants (*n* = 36) were interviewed by a registered dietitian nutritionist (RDN) to obtain a 24-hour diet recall, following the basic guidelines of the multiple-pass approach [22]. The recall was then analyzed using the web-based Automated Self-Administered 24-hour recall (ASA24-2016) for research, a web-based tool developed by researchers at the National Cancer Institute (NCI) [23] The participants completed a short dietary screening survey to assess routine calcium and vitamin D intake. The screening tool was developed and validated by researchers at Nutrition Quest using data from the National Health and Nutrition Examination Survey (NHANES) 1999–2001, which included 19 food items, three supplement questions, and questions to adjust for food fortification practices [24]

### Statistical analyses

Partial Spearman’s rank correlations were calculated to investigate the association between physiological variables and number of MetS risk factors. All correlations controlled for age and gender. Additional analyses were conducted, which independently controlled for HbA1c, VAT area, percent body fat, and physical fitness (exercise minutes per week and estimated VO_2max_). In calculating the ranked correlations, the number of MetS factors were categorized in bins of 0, 1, 2, and 3 or more risk factors. Odds ratios were calculated to d etermine the likelihood of a person having 3 or more MetS risk factors. In calculating odds ratios, number of metabolic syndrome risk factors were dichotomized to less than three and three or more. Other variables were dichotomized as follows, HbA1c elevated vs normal (≥5.7% vs <5.7%), VAT area elevated vs normal (≥100cm^2^ vs <100cm^2^), VO_2max_ above vs below the fiftieth percentile for age and gender, exercise minutes per week greater than or equal to 90 minutes per week vs less than 90 minutes per week, and percent body fat percentile above vs. below the fiftieth percentile for age, gender, and ethnicity. Kendall’s Tau correlations were used to investigate relationships between weight status and dietary variables of interest. Missing data were handled using pairwise deletion. The level of significance was set at p < 0.05. All analyses were conducted using SPSS version 24.0.

## Results

### Participants

The sample consisted of 106 participants, 43% female and 57% male, with a mean age of 46.8 years (Table 1). A sub-set of 36 participants completed dietary analysis and are included as a separate column in Table 1. Eighty-three percent of the participants identified as non-Hispanic White with the remainder identifying as Hispanic, African American, or Pacific Islander. All were non-smokers. Twenty-five percent of this sample were obese as indicated by a BMI of ≥ 30 kg/m^2^, 53% as overweight ≥ 25–29 kg/m^2^, and the remainder as normal weight. Fifty-four or 51% of participants had a VAT that placed them at increased risk for developing metabolic disease. Sixty-six or 63% of this sample self-reported weekly exercise of ninety or more minutes per week.

**Table 1:** Demographic characteristics of participants.

Demographic Data	Men n = 60	Women n = 46	Subset n = 36 (17 women, 19 men)
Age (years)	44.8 ± 14.6	49.5 ± 13.8	54.6 ± 14.5
Height (cm)	178.7 ± 7.7	164.4 ± 6.8	171.6 ± 11.7
Weight (kg)	90.4 ± 17.4	71.3 ± 11.8	83.2 ± 16.1
Waist Circumference (cm)	97.2 ± 14.0	88.9 ± 11.0	97.0 ± 12.6
Body Mass Index (kg/m^2^)			
Underweight (<18.5)	0	2	0
Normal (18.5–24.9)	15	14	8
Overweight (25–29.9)	28	22	19
Obese (≥30.0)	17	8	9
3 or more MetS Risk Factors	16	10	12
Prediabetes (HbA1c = 5.7–6.4%)	10	11	17
Type 2 Diabetes Mellitus (HbA1c = 6.5% or above)	3	1	3

Demographic data reported as mean ± standard deviation, all other data presented as counts. Subset group represents the thirty-six participants that also underwent a 24hr dietary recall.

Metabolic Syndrome, Prediabetes and T2DM. Twenty-six or 25% of participants had three or more risk factors for MetS; all with no previous diagnosis of MetS. In the sub-set of 36 participants as reported in a previous study, 14% had serum vitamin D deficiency or insufficiency as well as three or more risk factors for MetS [16]. Twenty-three or 22% of participants had two risk factors for MetS. The most common risk factor present in this sample was elevated fasting glucose (47%) followed by elevated blood pressure (43%), increased waist circumference (38%), elevated triglycerides (21%), and low HDL cholesterol (9%). Twenty-one participants (20%) had hgbA1c levels elevated at the pre-diabetes level with four in the diabetic range. All but one individual in the diabetic range had a previous diagnosis of type 2 diabetes, while those in the pre-diabetic range had no previous diagnosis.

### Correlation analysis

Partial correlations for number of MetS risk factors and physiologic variables are presented in Table 2. White blood cell count was significantly correlated with number of MetS risk factors (*r*_*s*_ = 0.522, *p <* 0.001). There was a significant correlation with HbA1c (*r*_*s*_ = 0.347, *p* < 0.01). The strength of this relationship was reduced and no longer significant when controlling for VAT area (*r*_*s*_ = 0.072, *p* > 0.05) but not when controlling for percent body fat (*r*_*s*_ = 0.299, *p* < 0.01).

**Table 2:** Correlation table.

Controlling for:		Age and gender	Age, gender, VO_2mas_, and weekly exercise minutes	Age, gender, and HbA1c	Age, gender, and VAT area	Age, gender, and percent body fat
Total Cholesterol	*r*_*s*_	-0.013	-0.040	-0.078	-0.015	0.006
	*p*	0.902	0.713	0.472	0.888	0.959
LDL Cholesterol	*r*_*s*_	-0.028	-0.066	-0.072	-0.044	-0.025
	*p*	0.796	0.544	0.510	0.686	0.817
White Blood Cell Count	*r*_*s*_	0.522*	0.417*	0.462*	0.365*	0.491*
	*p*	0.000	0.000	0.000	0.001	0.000
HbA_1C_	*r*_*s*_	0.347*	0.279*	NA	0.072	0.299*
	*p*	0.001	0.009	NA	0.507	0.005
Weekly Exercise Minutes	*r*_*s*_	-0.056	NA	0.009	0.155	0.083
	*p*	0.601	NA	0.938	0.151	0.447
VO_2max_	*r*_*s*_	-0.465*	NA	-0.411*	-0.190	-0.316*
	*p*	0.000	NA	0.000	0.077	0.003
Handgrip Strength	*r*_*s*_	0.006	-0.026	0.010	0.060	0.067
	*p*	0.959	0.814	0.928	0.582	0.536
BMI	*r*_*s*_	0.568*	0.425*	0.516*	0.249*	0.464*
	*p*	0.000	0.000	0.000	0.020	0.000
Fat Weight	*r*_*s*_	0.499*	0.292*	0.448*	0.033	0.341*
	*p*	0.000	0.006	0.000	0.761	0.001
Percent Body Fat	*r*_*s*_	0.390*	0.182	0.349*	-0.087	NA
	*p*	0.000	0.093	0.001	0.425	NA
Percent Android Fat	*r*_*s*_	0.463*	0.311*	0.393*	-0.073	0.283*
	*p*	0.000	0.004	0.000	0.499	0.008
Percent Gyndoid Fat	*r*_*s*_	0.326*	0.132	0.307*	-0.047	-0.071
	*p*	0.002	0.225	0.004	0.667	0.511
VAT Area	*r*_*s*_	0.592*	0.469*	0.515*	NA	0.489*
	*p*	0.000	0.000	0.000	NA	0.000

Partial Spearman’s Rho correlations between indicated variable and number of MetS risk factors.

*p < 0.05.

As markers of physical fitness, exercise time per week, estimated VO_2max_, and handgrip strength in relation to MetS risk factor number were investigated. Weekly exercise and handgrip strength were not significantly correlated to risk factor number. VO_2max_ was inversely related to MetS risk factors (*r*_*s*_ = -0.465, *p* < 0.001); however, this association was no longer statistically significant when controlling for VAT area (*r*_*s*_ = -0.190, *p* > 0.05).

A number of body composition variables were significantly correlated to MetS risk factor number: BMI (*r*_*s*_ = 0.568, *p* < 0.001), fat weight (*r*_*s*_ = 0.499, *p* < 0.001), percent body fat (*r*_*s*_ = 0.390, *p* < 0.001), percent android fat (*r*_*s*_ = 0.463, *p* < 0.001), percent gynoid fat (*r*_*s*_ = 0.326, *p* < 0.01), and VAT area (*r*_*s*_ = 0.592, *p* < 0.001). The relationship between BMI and MetS risk factors was reduced, when controlling for VAT area (*r*_*s*_ = 0.249, *p* < 0.05). Additionally, other body composition measures were no longer significantly correlated to MetS risk factor number: fat weight (*r*_*s*_ = 0.033, p > 0.05), percent body fat (*r*_*s*_ = -0.087, *p* > 0.05), percent android fat (*r*_*s*_ = -0.087, *p* > 0.05), percent gynoid fat (*r*_*s*_ = -0.047, *p* > 0.05). Controlling for PA mediated the relationship between body composition and MetS risk factor number with all correlation coefficients being reduced and percent body fat (*r*_*s*_ = 0.182, *p* > 0.05) and percent gynoid fat (*r*_*s*_ = 0.132, *p* > 0.05) no longer being significantly related to risk factor number.

### Odds ratios

Odds ratios were calculated to determine an individual’s likelihood of having three or more MetS risk factors. Elevated HbA1c (OR = 6.303, 95% CI: 2.290–17.346), elevated VAT area (OR = 20.000, 95% CI: 4.410–90.702), and being above the fiftieth percentile for percent body fat (OR = 9.899, 95% CI: 3.572–27.432) were all associated with an increased odds for MetS. Having a VO_2max_ above the 50^th^ percentile for age and gender was associated with a decreased odds for MetS (OR = 0.143, 95% CI: 0.048–0.423). Exercising for 90 minutes or more per week also decreased the odds for MetS (OR = 0.530, 95% CI: 0.215–1.310); however, this finding was not statistically significant. Odds ratios are presented in Table 3.

**Table 3:** Odds ratios.

	OR	95% Confidence Interval		n
		Lower	Upper	
HbA1c (Elevated vs. Normal)	6.303	2.290	17.346	100
VAT area (Elevated vs. Normal)	20.000	4.410	90.702	106
Percent body fat percentile (Above vs. Below 50^th^ percentile for age, ethnicity, and gender)	9.899	3.572	27.432	106
VO_2max_ (Above vs. Below 50^th^ percentile for age and gender)	0.143	0.048	0.423	102
Exercise per week (Above vs. Below 90 min. per week)	0.530	0.215	1.310	106

### Diet assessment

Though fiber intake was below levels recommended by the DGA [15] in the subset of subjects as a whole, significant correlations with fiber intake and weight status or any of the other lab values of interest were not identified. Two nutrients of interest, carbohydrate and fat, were correlated with BMI status. Percent kcal from carbohydrate intake (τ = -0.207, *p* < 0.05) had a negative correlation with BMI status and percent kcal from fat intake had a positive correlation (τ = 0.217, *p* < 0.05) with BMI status. Examining the relationship between diet variables and presence of metabolic risk factors revealed that fiber intake was significantly different between those with elevated waist circumference and those without (*p* < 0.05) and that percent of kcals coming from sugar was significantly different in individuals with elevated fasting triglycerides compared to those without (Table 4). Vitamin D intake was not associated with any MetS risk factors; however, serum vitamin D was negatively correlated to circulating triglycerides (τ = -0.273, *p* < 0.05). Similar frequencies of having three or more MetS risk factors were found between individuals with deficient levels of serum vitamin D (<20 ng/mL) and those with sufficient levels of serum vitamin D (≥20 ng/mL) (37.5% and 30.8%, respectively).

**Table 4:** Intake of select macro and micro nutrients [16].

% kcal Carbohydrate	46±10
% kcal Sugar	8±6
Fiber, gm	17±8
% kcal fat	36±9
% kcal saturated fat	11±4
% kcal protein	19±4
Calcium, mg, food & supplements	532±374
Vitamin D, IU, food & supplements	694±1202

## Discussion

This descriptive study evaluated the prevalence of MetS, prediabetes, and T2DM in a sample of participants undergoing a voluntary fitness assessment. Through the fitness assessments, recommendations to improve participants’ eating patterns, PA levels, and body composition were identified and provided to participants. The assessments and recommendations have the potential to improve participants’ overall health and well-being

Twenty-six or 25% of participants had three or more risk factors for MetS meeting diagnostic criteria [2]. This is lower than the 33% prevalence rate among U.S. adults as reported by Aguilar et al [1]. Twenty-two percent of participants had two risk factors placing them at risk for developing MetS in the future. Though blood pressure was measured in a non-clinical setting and therefore not diagnostic, 43% of participants had elevated blood pressure, higher than the national prevalence rate of 29%. Thirty-eight percent of participants had an elevated waist circumference; this finding is slightly lower than the prevalence rate of 56% from a study by Beltran-Sanchez et al [25] Interestingly in this sample of adults, total cholesterol and LDL cholesterol were not significantly correlated to any of the variables of interest, unlike findings from Holvoet, et al [26].

Having an elevated VAT area increased an individuals’ odds of having three or more risk factors for MetS by a magnitude of 20.000 (95% CI: 4.410–90.702) in this study. Additionally, many measurements of body composition were no longer significantly correlated to MetS risk factor number when controlling for differences in VAT area. Evidence supports visceral, versus subcutaneous adiposity is more detrimental to overall health and increases one’s risk of developing MetS, CVD, and T2DM [27] It is purported that the free fatty acids produced from the visceral adipocyte have portal drainage into the liver playing a significant role in the pathogenesis of MetS. This type of adipose tissue is more likely to produce inflammatory adipokines that are linked to insulin resistance [27]. As elevated fasting glucose is one of the risk factors for MetS, this potential link to insulin resistance is noteworthy [2].

White blood cell count is associated with pro-inflammatory cytokines, such as C-reactive protein and can be used as a measure of whole body inflammation [28]. Systemic inflammation is hypothesized to be a contributing factor to MetS [29]. Consistent with this idea, we report a strong correlation between white blood cell count and number of MetS risk factors.

In this sample, 20% of participants had HbA1c in the prediabetes range of 5.7–6.4%. This prevalence is slightly lower than the 2016 ADA report of 33.9% [13]. In the present study, an elevated HbA1c (5.7% and above) indicated that an individual was at significantly increased odds of having three or more risk factors of MetS, than an individual with normal HbA1c value. Three subjects had a previous diagnosis of T2DM and their corresponding fasting plasma glucose and HbA1c were indicative of this. One subject with no previous T2DM diagnosis, had both elevated fasting plasma glucose and HbA1c consistent with T2DM diagnostic criteria. Of these participants with HbA1c levels in the prediabetes range, approximately 44% would benefit from weight loss due to a BMI above 25 kg/m^2^. In a study by Shantha et al. [30], every 10% body weight reduction in overweight or obese individuals with T2DM there was a subsequent HbA1c percent reduction of 0.81. Losing weight to halt the progression of prediabetes to T2DM or as a treatment option for those with T2DM, would be beneficial in terms of both personal health and reduction of medical expenses.

A meta-analysis has indicated greater levels of leisure time PA are associated with a decreased risk for developing MetS [31]. Our results support this idea, finding that weekly exercise greater than ninety minutes a week was associated with a decreased odds of having three or more MetS risk factors, albeit this finding was not statistically significant. Additionally, in the present study, greater physical fitness, determined by having an estimated VO_2max_ above the fiftieth percentile for one’s age and gender, was significantly associated with decreased odds for having three or more MetS risk factors. These findings underscore not only the importance of PA, but also achieving an intensity of PA that is able to bring about improvements in cardiovascular fitness. The ACSM recommends adults aim for at least 150 minutes of moderate-intensity cardiorespiratory exercise each week and resistance train each major muscle group two to three days per week using a variety of equipment and/or exercises [8].

Diet variables of concern in this population match the aforementioned recommendations from the 2015–2020 DGA report [15]. The sub-group as a whole had inadequate fiber intake, inadequate calcium/vitamin D intake, and exceeded the recommendation for calories from added sugar and saturated fat (see Table 4). Protein intake was adequate across the group. The 2015–2020 DGA includes recommended consumption ratios of macronutrients. In this sub-group, almost one-third of participants had intakes below the recommended levels of kcal for carbohydrate and excess consumption of kcal from fat, including saturated fat. Carbohydrate containing foods include whole grains, fruits, and vegetables. These food groups are rich sources of many essential nutrients including fiber, therefore consuming adequate amounts is important to promote good health. Limiting saturated fat to less than 10% of total kcal is recommended as excess consumption is associated with reduced risk of CVD [15].

A review article by Slavin [32], outlines the many benefits of fiber intake in regards to body weight. The inverse association between fiber consumption and body weight and body fat has been established through a variety of studies [32] Adequate fiber intake has shown to be beneficial in regards to lowering of cholesterol levels [14]. Only two participants met the DGA recommended fiber intake, with an average of 17 grams per day for the sub-group. This is similar to findings from a ten-year trend review from the NHANES 2001–2010, where mean fiber intake for adults 19 years of age and older was approximately 16 grams per day [33]

Vitamin D and calcium intake can be correlated to consumption of dairy products. The 2015–2020 DGA reported inadequate intake of dairy products as a concern; this concern was also found among this small sample of participants [15]. When consumed in adequate amounts, calcium may increase fat oxidation, and when coupled with a restricted calorie diet, may promote fat loss [15,16]. Adequate intake of vitamin D may enhance the thermal effect of a meal and promote fat oxidation [34] Additionally, there is limited evidence that vitamin D may modify insulin sensitivity and in-turn control appetite, which may promote fat loss over time [35] For those individuals unable to consume adequate amounts of calcium and vitamin D through food intake, supplementation should be recommended, especially since food sources naturally containing vitamin D, not fortified are limited [15].

There are several limitations to this study. Firstly, this was a descriptive, cross-sectional study with a convenience sample size of mostly non-Hispanic White participants; this limits the generalizability to the general U.S. population. Using a 24-hour diet recall may have not given a true, routine estimate of dietary intake. As participants prepare to have a fitness assessment completed, they may have limited their routine food intake; however, the use of the vitamin D and calcium screener enhanced the ability to assess usual intake of these two specific nutrients.

## Conclusions

Findings from this small sample size of adults would indicate the need for routine screening for the clustering of MetS risk factors, prediabetes, and T2DM, in addition to treating these conditions. A majority of the participants would benefit from weight loss and a reduction in waist circumference to improve overall health outcomes. Increasing PA and therefore physical fitness in individuals not meeting current PA recommendations, would be beneficial as this is a known, modifiable risk factor for CVD. As the need for several shifts in food group consumption were identified, including nutrition education as part of the fitness assessment is necessary to improve eating patterns that can improve overall health. Making shifts towards a healthy eating pattern can help treat or prevent many chronic diseases to include those identified in this study.

## Supporting information

S1 File (XLSX)

S1 Survey (PDF)
