# Supplementary material for: Prevalence of metabolic disease and correlation to body composition and cardiovascular fitness in adults undergoing fitness assessments
Source: PLoS One. 2018 Dec 21;13(12):e0209514. doi: 10.1371/journal.pone.0209514 (PMC6303013; doi:10.1371/journal.pone.0209514)
Supplement: S1 Survey — (PDF) [file pone.0209514.s002.pdf]

ID NUMBER | AGE | SEX: Male / Female

If female, are you pregnant or breastfeeding? No / Yes

How often do you usually eat each of the foods listed below? Remember breakfast, lunch, dinner, snacks, and when eating out.

Please use a pencil.

© Copyright Nutritionquest. This form may not be used or reproduced without permission. Please call 510-704-8514 for reprints.

	HOW OFTEN IN THE PAST YEAR?						HOW MUCH ON THOSE DAYS?		
	Never	2-3 times per month	1-2 times per week	3-4 times per week	5-6 times per week	Every day			
Eggs or breakfast sandwiches with eggs	○	○	○	○	○	○			
Breakfast cereal, like corn flakes	○	○	○	○	○	○			
Oatmeal	○	○	○	○	○	○			
Orange juice	○	○	○	○	○	○	1 glass	2 glasses	3 glasses
Glasses of regular milk, soy milk, chocolate milk or cocoa (Don't count milk on cereal)	○	○	○	○	○	○	1 glass	2 glasses	3 glasses
Drinks like Sego, Slimfast, Slender or Ensure	○	○	○	○	○	○	1 glass	2 glasses	3 glasses
Breakfast bars, granola bars or energy bars like Power Bar, Slimfast Bar	○	○	○	○	○	○	1 bar	2 bars	3 bars
Bread, rolls or English muffins	○	○	○	○	○	○	1 slice	2 slices	3+ slices
Tortillas	○	○	○	○	○	○	1 tortilla	2 tortillas	3+ tortillas
Hamburger or cheeseburger with bun	○	○	○	○	○	○			
Pizza	○	○	○	○	○	○			
Tacos, burritos, enchiladas	○	○	○	○	○	○			
Macaroni & cheese, lasagna or cheese ravioli	○	○	○	○	○	○			
Pinto beans, black beans or chili with beans	○	○	○	○	○	○			
Cheese or cheese spread not counted above	○	○	○	○	○	○			
Yogurt	○	○	○	○	○	○			
Ice cream or frozen yogurt, regular or low-fat	○	○	○	○	○	○			
Cake or doughnuts	○	○	○	○	○	○			
Carbonated soft drinks, like Coke, ginger ale, orange soda or any kind (diet or regular)	○	○	○	○	○	○	1 glass	2 glasses	3 glasses
Tums or Rolaids	○	○	○	○	○	○	1 tablet per day	2 tablets per day	3 tablets per day
Multiple-vitamins, like One-a-Day, or prenatal	○	○	○	○	○	○			
Calcium supplements	○	○	○	○	○	○			
Vitamin D, alone or with calcium	○	○	○	○	○	○			

On the days that you take ***calcium supplements*** (Not including Tums/Rolaids) how many milligrams of calcium do you take **in the whole day?** ○ 300mg ○ 500mg ○ 600mg ○ 800mg ○ 1000mg ○ 1200mg ○ 1500mg ○ 1600mg

On the days you take ***vitamin D supplements*** (alone or combined with calcium) how many IU of vitamin D do you take **in the whole day?** ○ 200 IU ○ 400-500 IU ○ 600-800 IU ○ 1000 IU ○ 2000 IU ○ 5000 IU ○ 10,000+ IU

When you eat ***bread or rolls***, is it usually ○ Calcium-enriched ○ Not calcium-enriched ○ Don't know

When you drink ***orange juice***, is it usually ○ With added calcium ○ Without added calcium ○ Don't know

When you eat ***burgers*** are they usually ○ Hamburgers ○ Cheeseburgers ○ Both equally

When you eat ***cold cereal*** is it usually ○ "Total" brand cereal ○ Some other kind of cereal
